# Alpha-Mangostin: A Potent Inhibitor of TRPV3 and Pro-Inflammatory Cytokine Secretion in Keratinocytes

**DOI:** 10.3390/ijms241612930

**Published:** 2023-08-18

**Authors:** Thi Huyen Dang, Ji Yeong Kim, Hyun Jong Kim, Byung Joo Kim, Woo Kyung Kim, Joo Hyun Nam

**Affiliations:** 1 Department of Physiology, Dongguk University College of Medicine, Gyeongju 38066, Republic of Korea; danghuyenhup@gmail.com (T.H.D.); designed_hj@naver.com (H.J.K.); 2 Channelopathy Research Center (CRC), Dongguk University College of Medicine, Goyang 10326, Republic of Korea; 3 Department of Physiology, Ischemic/Hypoxic Disease Institute, Seoul National University College of Medicine, Seoul 03080, Republic of Korea; lotuskim@snu.ac.kr; 4 Department of Longevity and Biofunctional Medicine, Pusan National University School of Korean Medicine, Yangsan 50612, Republic of Korea; vision@pusan.ac.kr; 5 Department of Internal Medicine Graduate School of Medicine, Dongguk University, Goyang 10326, Republic of Korea

**Keywords:** keratinocytes, alpha-mangostin, TRPV3, skin inflammation, dermatitis

## Abstract

The TRPV3 calcium ion channel is vital for maintaining skin health and has been associated with various skin-related disorders. Since TRPV3 is involved in the development of skin inflammation, inhibiting TRPV3 could be a potential treatment strategy. Alpha-mangostin isolated from *Garcinia mangostana* L. extract exhibits diverse positive effects on skin health; however, the underlying mechanisms remain obscure. This study investigated the TRPV3-inhibitory properties of alpha-mangostin on TRPV3 hyperactive mutants associated with Olmsted syndrome and its impact on TRPV3-induced cytokine secretion and cell death. Our findings demonstrate that alpha-mangostin effectively inhibits TRPV3, with an IC_50_ of 0.077 ± 0.013 μM, showing inhibitory effects on both wild-type and mutant TRPV3. TRPV3 inhibition with alpha-mangostin decreased calcium influx and cytokine release, protecting cells from TRPV3-induced death. These results indicate that alpha-mangostin reduced inflammation in TRPV3-activated skin keratinocytes, suggesting that alpha-mangostin could be potentially used for improving inflammatory skin conditions such as dermatitis.

## 1. Introduction

The growth and development of keratinocytes and the establishment and maintenance of skin barriers and other skin processes depend highly on the distribution of Ca^2+^ throughout the epidermis [1]. Both intracellular and extracellular concentrations of Ca^2+^ have been reported to modulate various functions in the skin, suggesting the involvement of functional calcium channels expressed in epidermal cells [1].

Among these channels, the Ca^2+^-permeable non-selective cation channel TRPV3 is highly expressed in epidermal keratinocytes [2]. The activity of TRPV3 channels regulates important processes such as keratinocyte growth [3]; barrier formation [4]; wound healing [5]; hair growth [6]; and the perception of temperature, itch, and pain [7,8]. Studies on hairless mice with TRPV3 gain-of-function mutations (G573C in WBN/Kob-Ht and G573S in DS-Nh mice) have highlighted the relevance of TRPV3 in both normal and abnormal skin conditions [9]. These mutations have been associated with hair loss, dermatitis, and pruritus [10], and similar mutations also appeared in humans with a skin inflammatory condition called Olmsted syndrome [11]. Additionally, TRPV3 upregulation has been observed in mouse models of atopic dermatitis (AD) and inhibition of TRPV3 channel activity has shown promise in attenuating AD symptoms [12,13]. Activation of TRPV3 in keratinocytes leads to the release of interleukins and other inflammatory factors, contributing to skin inflammation and itch [14,15,16,17]. Therefore, identifying novel TRPV3 inhibitors holds the potential for developing new strategies to improve skin inflammation and pruritus.

Historically, natural medicines have served as valuable candidates for drug development [18]. Many prescription drugs developed over the past decades for health improvement and disease treatment contain active compounds derived from medicinal plant extracts [19]. *Garcinia mangostana* L., commonly known as mangosteen, has gained significant attention due to its biological activities in various diseases. One of its compounds, alpha-mangostin, derived from the pericarps of mangosteen fruit, has demonstrated anti-cancer, anti-diabetic, anti-bacterial, anti-inflammatory, and anti-oxidative properties with promising anti-inflammatory effects [20,21]. Ointments containing mangosteen peel have been utilized to treat conditions such as eczema and skin malfunctions, and for wound healing [22]. A study also revealed that mangosteen rind extract could prevent AD by controlling inflammation and itch, and improving skin barrier function [23]. Moreover, alpha-mangostin has been found to promote wound healing and suppress cytokine expression in HaCaT cells stimulated by *P. acnes* and UVB [24,25].

Here, we found that alpha-mangostin inhibited human TRPV3 currents in a dose-dependent manner, decreased calcium influx, reduced cell death caused by aberrant TRPV3 activity, and suppressed carvacrol-induced cytokine secretion in human keratinocytes. These findings indicate that alpha-mangostin could be developed as a drug to improve skin conditions.

## 2. Results

### 2.1. Alpha-Mangostin Significantly Inhibits TRPV3 Current

**Figure 1:**
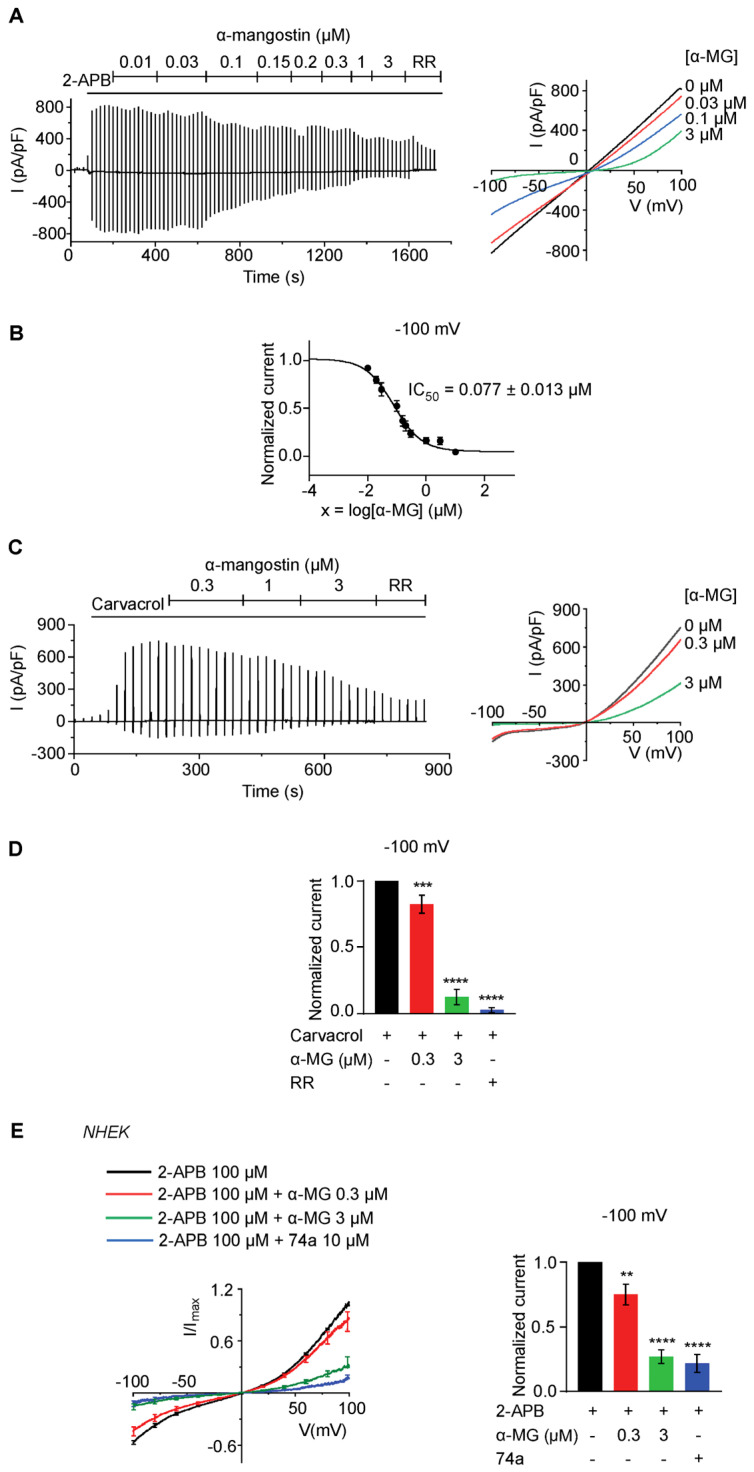
Alpha-mangostin (α-MG) inhibited the TRPV3 current. Currents were elicited with ramp pulses from −100 to +100 mV over 1 s, which was repetitively applied every 20 s; the holding potential was set at 0 mV. (**A**) Alpha-mangostin inhibited 2-APB-evoked current in a representative TRPV3 overexpressing HEK 293T cell. The channel was activated using 100 µM 2-APB and subsequently perfused with solutions containing different concentrations of alpha-mangostin with 100 µM 2-APB. A solution containing 10 µM ruthenium red (RR, a TRP channel inhibitor) and 100 µM 2-APB was perfused at the end. (**B**) The dose–response curve of TRPV3 current inhibition by alpha-mangostin (IC_50_ = 0.077 ± 0.013 µM). Currents were normalized to the maximum response to 2-APB at −100 mV. (**C**) Alpha-mangostin inhibited carvacrol-evoked current in a representative TRPV3-overexpressing HEK 293T cell. TRPV3 was activated using 1000 µM carvacrol and subsequently perfused with solutions containing different concentrations of alpha-mangostin with 1000 µM carvacrol. A solution containing 10 µM RR and 1000 µM carvacrol was perfused at the end. (**D**) Summary of normalized currents evoked by carvacrol with or without alpha-mangostin at −100 mV. Currents were normalized to the maximum response carvacrol at −100 mV. (**E**) Alpha-mangostin inhibited 2-APB-evoked current in NHEK. The channel was activated using 100 µM 2-APB and subsequently perfused with solutions containing different concentrations of alpha-mangostin with 100 µM 2-APB. A solution containing 10 µM 74a (a TRPV3 inhibitor) and 100 µM 2-APB was perfused at the end. The left panel shows the current–voltage relationship; I_max_ represents the maximum response to 100 µM 2-APB at +100 mV. The right panel summarizes normalized currents evoked by 2-APB with or without alpha-mangostin at −100 mV. Currents were normalized to the maximum response to 2-APB at −100 mV. Data are presented as means ± SEM. ** *p* < 0.01, *** *p* < 0.001, **** *p* < 0.0001.

First, we used a conventional whole-cell patch-clamp to record the TRPV3 current in HEK 293T cells overexpressing hTRPV3. Then, different concentrations of alpha-mangostin were perfused into the cells to determine the effects of alpha-mangostin on the TRPV3 current. Alpha-mangostin inhibited 2-APB-evoked TRPV3 current, as shown in a representative cell in Figure 1A. Alpha-mangostin presented remarkable dose-dependent inhibitory effects on 2-APB-induced TRPV3 current with an IC_50_ of 0.077 ± 0.013 μM at −100 mV (Figure 1B), and its inhibitory effect was partly reversible (Supplementary Figure S1B). At 10 µM, alpha-mangostin almost completely inhibited the 2-APB-evoked currents to a similar level to that of ruthenium red, a broad-spectrum TRP inhibitor (Supplementary Figure S1A). Alpha-mangostin also suppressed the TRPV3 current induced by another agonist, carvacrol, in TRPV3-overexpressing HEK 293T cells at higher concentrations (Figure 1C,D).

In consideration of the high expression level and function of TRPV3 in keratinocytes [26], we examined the impact of alpha-mangostin on 2-APB-evoked TRPV3 current in normal human epidermal keratinocytes (NHEK). On average, alpha-mangostin at 0.3 and 3 µM inhibited TRPV3 currents to 75.14% ± 7.94% and 27.03% ± 5.25%, respectively (Figure 1E).

### 2.2. Alpha-Mangostin Is a Potent TRPV3 Inhibitor

**Figure 2:**
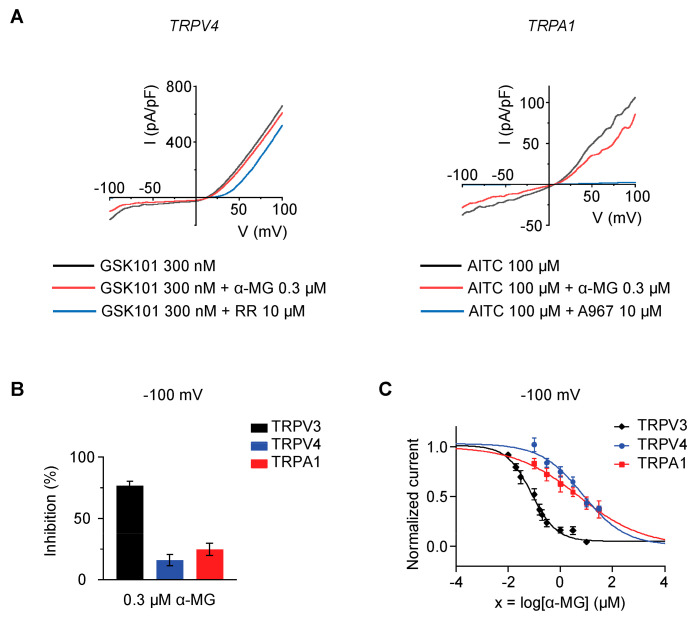
Alpha-mangostin (α-MG) inhibited the TRPV3 current more effectively than other TRP channels. (**A**) Representative I–V curves from whole-cell recordings illustrate the inhibitory effect of 0.3 µM alpha-mangostin on TRPV4 and TRPA1 channels (GSK101: GSK-1016790A, a TRPV4 agonist; RR: ruthenium red, a TRP inhibitor; AITC: allyl isothiocyanate, a TRPA1 agonist; A967: A-967079, a TRPA1 inhibitor). Currents were elicited with ramp pulses from −100 to +100 mV over 1 s, which were repetitively applied every 20 s; the holding potential was set at 0 mV. (**B**) Summary of the inhibition percentage after 0.3 µM alpha-mangostin treatment at −100 mV. (**C**) The dose–response curve of indicated channel currents by alpha-mangostin in comparison with TRPV3 from Figure 1A (IC_50_ = 0.077 ± 0.013 µM, IC_50_ = 8.470 ± 1.725 µM, and IC_50_ = 5.092 ± 2.213 µM for TRPV3, TRPV4, and TRPA1, respectively).

Next, we examined the impact of alpha-mangostin on other TRP channels. TRPV4- and TRPA1 trasfected HEK 293T were activated using 300 nM GSK-1016790A (GSK101) and 100 µM allyl isothiocyanate (AITC), respectively. The presence of 0.3 µM alpha-mangostin slightly inhibited TRPV4 and TRPA1 currents (16.08% ± 4.62% for TRPV4 and 24.77% ± 4.97% for TRPA1) but effectively inhibited TRPV3 currents (76.60% ± 3.55%) (Figure 2A,B). The dose–response curves were obtained by applying various concentrations of alpha-mangostin (Figure 2C). Alpha-mangostin showed much weaker inhibitory effects on TRPV4 and TRPA1 currents (IC_50_ = 8.470 ± 1.725 µM for TRPV4 and IC_50_ = 5.092 ± 2.213 µM for TRPA1) in comparison with TRPV3 (IC_50_ = 0.077 ± 0.013 µM), implying that alpha-mangostin is a potent TRPV3 inhibitor.

### 2.3. Alpha-Mangostin Noticeably Inhibits Ca^2+^ Influx Mediated through TRPV3 Channels

**Figure 3:**
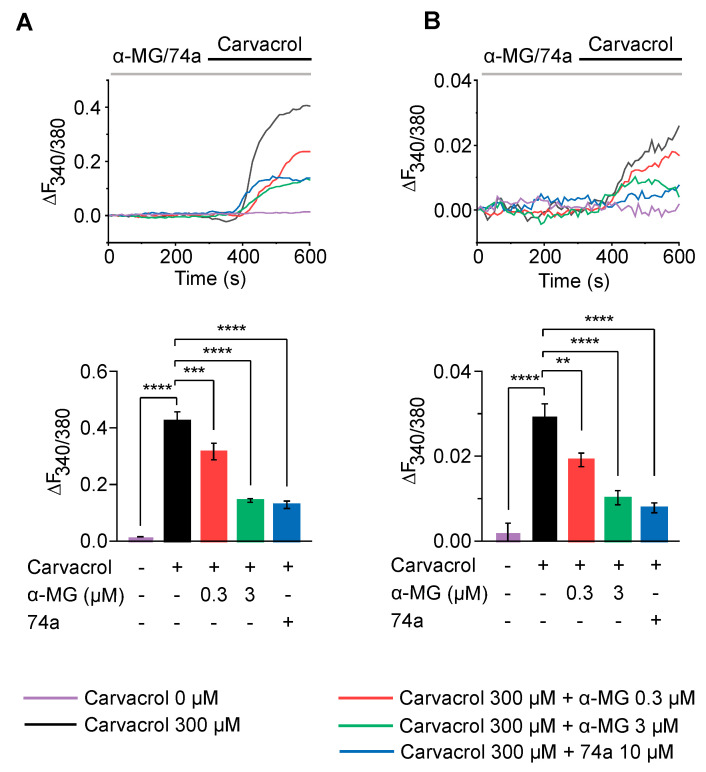
Alpha-mangostin (α-MG) inhibited TRPV3-mediated intracellular calcium increase. Cells were perfused with solutions containing 0.3 µM, 3 µM alpha-mangostin or 10 µM 74a before the addition of 300 µM carvacrol (red, green, and blue lines, respectively). Cells only perfused with normal Tyrode (NT) solution in the presence (black line) or absence (purple line) of 300 µM carvacrol were employed as the control. (**A**) Alpha-mangostin inhibited TRPV3-mediated intracellular calcium increase in response to carvacrol in TRPV3-overexpressing HEK 293T cells. The top panel shows a representative trace. The bar graph on the bottom summarizes the change in the intracellular calcium rise inhibited by alpha-mangostin. (**B**) Alpha-mangostin inhibited TRPV3-mediated intracellular calcium increase in response to carvacrol in normal human epidermal keratinocytes (NHEK). The top panel shows a representative trace. The bar graph on the bottom summarizes the change in the intracellular calcium rise inhibited by alpha-mangostin. Data are presented as means ± SEM. ** *p* < 0.01, *** *p* < 0.001, **** *p* < 0.0001.

Since the TRPV3-mediated calcium influx is reported to contribute to proper skin function [4,27], we investigated whether alpha-mangostin inhibits the intracellular calcium increase induced by carvacrol in hTRPV3-overexpressing HEK 293T cells and keratinocytes. As shown in Figure 3, adding carvacrol triggered significant Ca^2+^ influxes, which were remarkably reduced by alpha-mangostin in both cell lines. In hTRPV3-overexpressing HEK 293T cells, ∆(F_340_/F_380_) decreased by approximately 25% and 70% upon exposure to 0.3 µM and 3 µM alpha-mangostin, respectively (Figure 3A, 0.424 ± 0.032 vs. 0.316 ± 0.030 and 0.144 ± 0.006, respectively). Similar effects were observed in keratinocytes, in which the change in F_340_/F_380_ was also smaller in alpha-mangostin-treated groups compared with the control ones (Figure 3B, alpha-mangostin 0.3 µM: 0.019 ± 0.002, alpha-mangostin 3 µM: 0.010 ± 0.002 vs. 0.029 ± 0.003 in the control group).

### 2.4. Alpha-Mangostin Inhibits Channel Activity and Rescues HEK 293T Cells Transfected with TRPV3 Mutations (G573S, G573C) from Cell Death

Previous studies demonstrated that point mutations G573S and G573C in TRPV3 caused constitutive channel opening, resulting in calcium overload and cell death [9,11]. To determine whether alpha-mangostin inhibits the activity of the mutated channel, we developed HEK 293T cells transiently expressing either wild-type (WT) or mutated (G573S, G573C) TRPV3.

**Figure 4:**
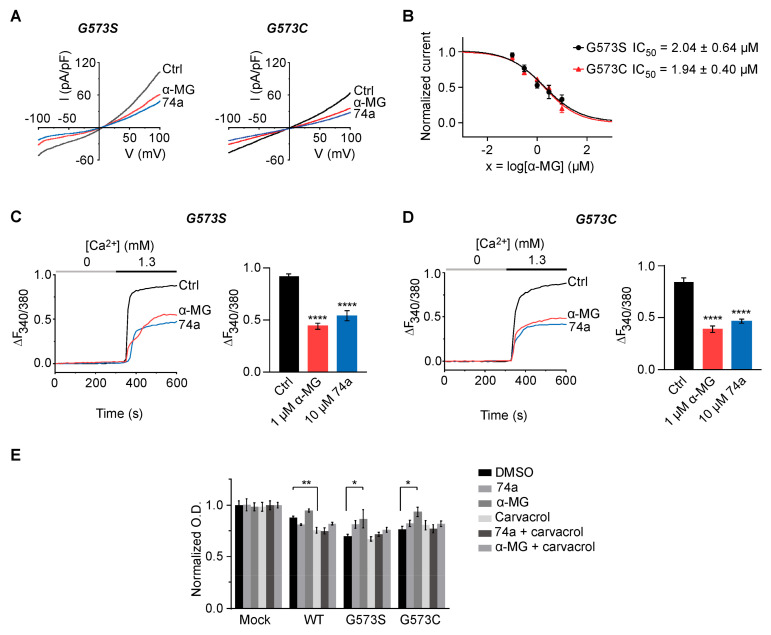
Alpha-mangostin inhibited the activity of hyperactive TRPV3 mutant channels and rescued mutation-induced cell death. (**A**) Alpha-mangostin inhibited the channel current in representative TRPV3 (G573S)- and TRPV3 (G573C)-expressing HEK 293T cells. (**B**) The dose–response curve of alpha-mangostin in cells expressing the TRPV3 mutants (IC_50_ = 2.04 ± 0.64 µM for G573S and IC_50_ = 1.94 ± 0.40 µM for G573C). Currents were normalized to the response at −100 mV before adding alpha-mangostin. (**C**) Alpha-mangostin inhibited TRPV3-mediated intracellular calcium increase in TRPV3 (G573S)-expressing HEK 293T cells. The left panel shows a representative trace. The bar graph on the right summarizes the change in the intracellular calcium rise inhibited by alpha-mangostin. Cells were perfused with calcium-free solutions containing 0 or 1 µM alpha-mangostin or 10 µM 74a before changing to 1.3 mM Ca^2+^ solutions containing the same substance concentration. (**D**) Alpha-mangostin inhibited TRPV3-mediated intracellular calcium increase in TRPV3 (G573C)-expressing HEK 293T cells. The left panel shows a representative trace. The bar graph on the right summarizes the change in the intracellular calcium rise inhibited by alpha-mangostin. Cells were perfused with calcium-free solutions containing 0 or 1 µM alpha-mangostin or 10 µM 74a before changing to 1.3 mM Ca^2+^ solutions containing the same substance concentration. (**E**) Alpha-mangostin improved cell viability in TRPV3 (G573S)- and TRPV3 (G573C)-expressing HEK 293T cells. Transfected HEK 293T cells were plated onto a 96-well plate 6 h after transfection and treated with 300 µM carvacrol, 1 µM alpha-mangostin, 10 µM 74a, or a mixture of 300 µM carvacrol and either 1 µM alpha-mangostin or 10 µM 74a for 24 h before CCK-8 assays. The optical density (O.D.) values were normalized to the untreated HEK 293T cells. Data are presented as means ± SEM. * *p* < 0.05, ** *p* < 0.01, **** *p* < 0.0001.

For electrophysiology recording and calcium measurement, 10 µM 74a (a TRPV3 inhibitor) was added about 6 h after transfection to inhibit channel activities and prevent cell death. First, the electrophysiology activities of the mutations were recorded using a whole-cell patch-clamp. Our data presented spontaneous TRPV3 currents when −100 mV to 100 mV ramp pulses were applied, which dramatically declined with the addition of 1 µM alpha-mangostin (Figure 4A). Alpha-mangostin inhibited the spontaneous current of TRPV3 channels with both G573 mutants in a dose-dependent fashion, with IC_50_ values of 2.04 ± 0.64 µM and 1.94 ± 0.40 µM for the G573S and G573C mutants, respectively (Figure 4B).

Subsequently, the effects of alpha-mangostin on the activities of the mutated channels were further evaluated using intracellular calcium measurements. Consistent with the patch-clamp data, a spontaneous intracellular calcium increase was observed in HEK 293T cells expressing gain-of-function mutations, which were potently attenuated by 1 µM alpha-mangostin (Figure 4C,D).

Next, we examined whether alpha-mangostin could rescue cell death caused by these aberrant mutant channels. Transfected cells were exposed to 300 µM carvacrol, 1 µM alpha-mangostin, 10 µM 74a, or the combination of carvacrol and either alpha-mangostin or 74a for 24 h before measuring the cell viability using CCK-8. As presented in Figure 4E, compared with mock cells, the viability in G573S- and G573C-transfected cells was 69.69% ± 1.86% and 76.19% ± 3.40%, respectively, which was lower than in the WT group (87.73% ± 1.80%). The viabilities of the cells carrying the mutated channels were significantly increased in the presence of alpha-mangostin (G573S: 86.77% ± 8.8%, G573C: 93.55% ± 4.51%, respectively). The cell-protective effects of alpha-mangostin were similar to that of 74a, a selective TRPV3 inhibitor. Although alpha-mangostin increased the viability of cells expressing the WT channel, it was not statistically significant. However, carvacrol treatment reduced the viability of cells expressing the WT TRPV3 to 75.62% ± 2.62%, which was reversed by alpha-mangostin, 82.07% ± 1.34%.

### 2.5. Alpha-Mangostin Inhibits Cytokine Release and Rescues Keratinocytes from Cell Death Caused by TRPV3 Agonists

**Figure 5:**
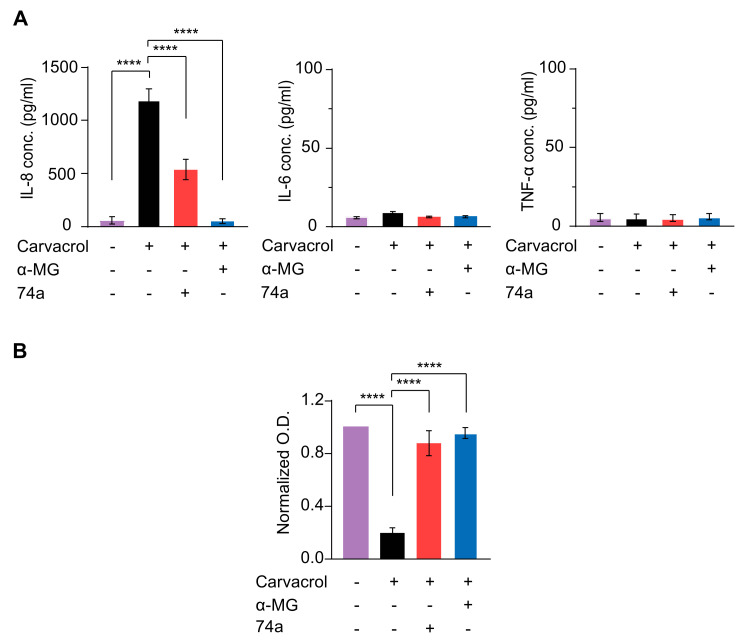
Alpha-mangostin (α-MG) inhibited carvacrol-induced cytokine release in NHEK cells and rescued carvacrol-induced cell death. (**A**) Alpha-mangostin inhibited the carvacrol-induced release of IL-8 and IL-6 in NHEK. Cells were induced by 300 µM carvacrol with or without either 1 µM alpha-mangostin or 10 µM 74a for 24 h before the ELISA assay. (**B**) Alpha-mangostin reduced cell death caused by carvacrol in human keratinocytes. Cells were induced by 500 µM carvacrol with or without either 0.3 µM alpha-mangostin or 10 µM 74a for 24 h. The optical density (O.D.) values were normalized to those of the untreated cells. Data are shown as means ± SEM. **** *p* < 0.0001.

Constitutive activation of TRPV3 due to continuous stimulation or the presence of Olmsted mutations causes noticeable skin inflammation due to the increased secretion of pro-inflammatory cytokines from the keratinocytes [15]. Besides, in atopic dermatitis (AD), cytokine release from keratinocytes contributes to the inflammatory environment, which drives many characteristic AD symptoms [28]. Therefore, we determined the effect of TRPV3 activation on cytokine release instead of TRPV3 expression and evaluated whether alpha-mangostin suppressed TRPV3-mediated cytokine secretion in normal human keratinocytes. The levels of secreted cytokines (interleukin (IL)-6, IL-8, and tumor necrosis factor-alpha (TNF-α)) were measured using enzyme-linked Immunosorbent assay (ELISA) kits due to their high sensitivity and quantitation ability. The cells were induced with 300 µM carvacrol, with or without 1 µM alpha-mangostin, and 10 µM 74a. After 24 h of incubation, carvacrol significantly stimulated IL-8 release but had a non-significant effect on TNF-α levels (Figure 5A). Treatment with alpha-mangostin or 74a drastically attenuated the IL-8 levels and slightly decreased the IL-6 levels, while no changes in TNF-α levels were observed (Figure 5A).

Strong activation of TRPV3 in keratinocytes by carvacrol at concentrations higher than 300 µM was reported to cause cell death [3]. Therefore, we examined the effect of alpha-mangostin in keratinocytes exposed to 500 µM carvacrol. As shown in Figure 5B, 500 µM carvacrol drastically decreased cell viability, which was significantly reversed by alpha-mangostin or the TRPV3 inhibitor, 74a (Figure 5B).

## 3. Discussion

TRPV3 is a cation non-selective, Ca^2+^-preferable channel and a member of the TRP superfamily [29]. TRPV3, abundantly found in epithelial cells, probably is the most essential TRPV channel to skin physiology and pathophysiology [26] and participates in various processes, including skin barrier formation [4], hair cycle regulation [6], and wound healing [5]. The level of TRPV3 mRNA and/or protein in skin cells was reported to be altered in several conditions, such as dermatitis [16,30,31], suggesting its potential role in the development of skin conditions [32]. Moreover, excessive TRPV3 activity was shown to be associated with inflammation and itches in Olmsted syndrome [10,11,16]. Thus, inhibiting TRPV3 activity might be beneficial in improving inflammatory skin conditions.

This study identified alpha-mangostin as a potent TRPV3 inhibitor with an IC_50_ of 0.077 ± 0.013 μM, as determined through patch-clamp recordings combined with calcium measurements. Alpha-mangostin exhibited stronger inhibitory effects on TRPV3 compared with other TRP channels: TRPV1 (IC_50_ = 0.43 ± 0.27 µM) [33], TRPV4 (IC_50_ = 8.470 ± 1.725 µM), and TRPA1 (IC_50_ = 5.092 ± 2.213 µM) (Figure 2C). Several natural compounds have been reported to inhibit TRPV3 channel activity; however, some of them show similar IC_50_ values on many targets (monachomycalin B; pulchranin A, B, C), while the others need to be used at higher concentrations (citrusinine II, vervacoside, coumarin osthole) (Table 1). Moreover, alpha-mangostin inhibited both WT TRPV3 and the TRPV3 gain-of-function mutants, suggesting its potential use for treating skin diseases. Natural TRPV3 inhibitors exhibit various chemical structures, implicating different molecular mechanisms for the inhibition effects. Among them, alpha-mangostin presents some structural similarities with citrusinine II, both including aromatic rings with hydroxyl group substitutions. Citrusinine inhibits the TRPV3 channel via the direct interaction of the benzene ring with the Y564 residue in the S4 helix [34]. This suggests that xanthone or acridone derivatives might be a promising source of TRPV3 inhibitors. However, the molecular mechanism underlying the TRPV3 inhibitory effect of alpha-mangostin is still unclear and requires further investigation.

The activation of TRPV3 leads to increased intracellular calcium levels and triggers various signaling pathways that regulate cell functions [26]. Activation of TRPV3 with its agonist, carvacrol, has been reported to induce calcium influx and decrease cell viability and proliferation by inducing apoptosis [15]. Higher [Ca^2+^]_i_ levels with elevated apoptosis and skin hyperkeratosis were also reported to be associated with TRPV3 gain-of-function mutations [11,15]. In line with these results, we found that stimulating TRPV3 with an agonist in WT-TRPV3-expressing cells increased intracellular calcium levels and decreased cell viability. In addition, our results demonstrated that stimulating TRPV3 increased intracellular calcium levels and decreased cell viability in both WT and Olmsted mutant TRPV3-expressing cells (Figure 3, Figure 4 and Figure 5). However, treatment with alpha-mangostin attenuated these effects by reducing intracellular calcium levels and improving cell viability in both cell types (Figure 3, Figure 4 and Figure 5).

Furthermore, activation of TRPV3 by heat or chemical stimuli was reported to induce pro-inflammatory responses in keratinocytes, including the upregulation of several cytokines, such as IL-8, IL-6, and TNF-α [15]. In our study, activating the TRPV3 channel using carvacrol significantly increased IL-8 secretion. Although a slight increase in IL-6 was observed, it was not significant; no changes in TNF-α secretion were observed. In addition, inhibiting TRPV3 using alpha-mangostin attenuated the increased IL-8 and IL-6 secretion in carvacrol-induced keratinocytes (Figure 5).

*Garcinia mangostana* (mangosteen) extracts have been used as a drug for various diseases for hundreds of years, including several skin infections, eczema, hyperkeratosis, and wounds [20]. A previous study showed that mangosteen rind extract suppressed skin inflammation, keratinocyte proliferation, and itch sensation in an AD mouse model, suggesting its protective potential in the early phase of AD [23]. According to our results, this effect could be attributed to the inhibition of TRP channels, especially TRPV3, on skin keratinocytes by alpha-mangostin, the primary xanthone obtained from mangosteen pericarp, as there is a clear relationship between TRPV3 and dermatitis [42]. Moreover, applying alpha-mangostin nanoparticles in patients with acne vulgaris significantly improved the skin condition [43]. Since the inhibition of the TRPV3 channel is expected to result in normalizing keratinocyte proliferation and inhibiting pro-inflammatory mediators in acne vulgaris [26], the inhibitory effects of alpha-mangostin on TRPV3 and cytokine release in NHEK could play important roles in acne-related skin conditions. Besides the anti-inflammatory effect, mangosteen pericarp extract was reported to promote hair growth in vitro [44], which could be attributed to the inhibition of TRPV3 channels expressed in hair follicles as the activation of TRPV3 was shown to regulate hair growth negatively [26]. The results from the current study demonstrate for the first time that the skin and hair health-improving effect of alpha-mangostin and *G. mangostana* extract is mediated through the inhibition of TRP channels, especially TRPV3.

There are some limitations in our study. First, in the whole-cell patch-clamp experiments, we used ruthenium red, a non-selective TRP channel inhibitor, to obtain the leak current; nevertheless, other TRP currents can be excluded due to the use of the TRPV3-overexpressing HEK 293T cell line. Further, calcium imaging but not patch-clamp measurement was used to assess the impact of alpha-mangostin on TRPV3 channel activity in keratinocytes; therefore, carvacrol and a more specific TRPV3 inhibitor, 74a, were used in these experiments. Moreover, we used the CCK8 assay to measure cell viability instead of apoptosis measurement to investigate the ability to rescue Olmsted-mutant-induced cell death.

## 4. Materials and Methods

### 4.1. Chemicals

2-aminoethoxydiphenyl borate (2-APB), carvacrol, GSK-1016790A (GSK101), allyl isothiocyanate (AITC), A-967079 (A967), ruthenium red (RR), and α-mangostin (α-MG) were purchased from Sigma-Aldrich (Saint Louis, MO, USA); 74a was purchased from Tocris Bioscience (Ellisville, MO, USA). Stock solutions, including 100 mM 2-APB, 300 mM and 1 M carvacrol, 300 µM GSK101, 100 mM AITC, 10 mM A967, 10 mM ruthenium red, 10 mM 74a, and 1 mM and 3 mM α-mangostin, were prepared in DMSO and stored in a freezer (−20 °C).

### 4.2. Cell Culture

NHEK (Catalog No. 00192627, Lonza, Basel, Switzerland) were grown in Keratinocyte Growth medium BulletKitTM (KGMTM, Lonza) at 37 °C in a 5% CO_2_ incubator and sub-cultured when they reached at least 70% confluence.

HEK 293T cells were purchased from the American Type Culture Collection (Cat. No. CRL-3216, ATCC, Manassas, VA, USA). TRPV3-overexpressing HEK 293T cells were supplied by Prof. Wan Lee (Dongguk University College of Medicine, Gyeongju, South Korea). HEK 293T cells expressing TRPV3 mutants (G573S, G573C), TRPV4, TRPA1 were made by transient transfection. These cells were cultured in Dulbecco’s modified Eagle’s medium (DMEM, Welgene, Daegu, Korea) containing 10% fetal bovine serum (FBS; Welgene) and 1% penicillin/streptomycin (P/S; Life Technologies, Carlsbad, CA, USA). Blasticidin S (Invitrogen, Carlsbad, CA, USA; 10 µg/mL) was used as the selection antibiotic for TRPV3-overexpressing HEK 293T cells. Cells were incubated at 37 °C in a 10% CO_2_ incubator.

### 4.3. Construct of cDNA and Transfection

Plasmids carrying TRPV3 (pReceiver-M02), TRPV4 (pReceiver-M29), and TRPA1 (pReceiver-M29) were bought from Genecopoeia (Rockville, MD, USA). Using the Quick Change II XL Site-Directed Mutagenesis Kit (Agilent Technologies, Santa Clara, CA, USA), mutant TRPV3 channels were generated using the PCR-based site-directed mutagenesis method. The forward and reverse primers for the G573S mutant were 5′-GGGGTTTCCAGTCCATGAGCATGTACAGCGTCATG-3′ and 5′-CATGACGCTGTACATGCTCATGGACTGGAAACCCC-3′, respectively. The forward and reverse primers for the G573C mutant were 5′-GGGGTTTCCAGTCCATGTGCATGTACAGCGTCATG-3′ and 5′-CATGACGCTGTACATGCACATGGACTGGAAACCCC-3′, respectively. Sequencing was used to confirm mutagenesis.

TRPV3 (WT and mutants), TRPV4, and TRPA1 plasmids were transiently transfected into HEK 293T cells, as previously described [45,46]. pEGFP (pEGFP-N1, Life Technologies) was co-transfected with the plasmid carrying TRPV3 WT or the TRPV3 mutants into HEK 293T cells to identify transfected cells. Turbofect reagent (Thermo Fisher Scientific, Waltham, MA, USA) was used for transfection. All experiments were performed after a further 24–36 h incubation after transfection.

### 4.4. Electrophysiological Recording

An Axopatch 200A amplifier and Digidata 1440A digitizer (Molecular Devices, Sunnyvale, CA, USA) were used for patch-clamp recording, as described previously [46,47].

The composition of the bath solution and pipette solution used to measure TRPV3, TRPV4, and TRPA1 current are presented in Table 2.

In the whole-cell configuration, voltage ramps from −100 to +100 mV over 1 s were applied every 20 s and the holding potential was set at 0 mV. Sampling at 10 kHz and low-pass filtering at 5 kHz (w-c) were applied for all recorded currents. Recorded data were analyzed using Clampfit ver. 10.7 (Molecular Devices) and Origin 2021b (Microcal, Northampton, MA, USA).

### 4.5. Calcium Imaging

Fura-2 AM (Thermo Fisher Scientific) was utilized as a fluorescent indicator to measure calcium signal, as described previously [48]. Fura-2 acetoxymethyl ester (Fura-2 AM) is a widely used fluorescent calcium indicator. After crossing the cell membrane and entering the cytosol, Fura-2 AM is cleaved into its activated form, Fura-2 [49]. After binding to the cytosolic free Ca^2+^, the peak excitation of Fura-2 shifts from 380 nm (Ca^2+^-free state) to 340 nm (Ca^2+^-bound state), while the peak emission remains unchanged at around 510 nm. To measure the cytosolic Ca^2+^ level, a sequential excitation of Fura-2 is performed at 340 nm and 380 nm; then, the emission signals resulting from each excitation wavelength are measured and the ratio of these signals is calculated. By comparing this emission ratio with the emission ratios obtained from known concentrations of free Ca^2+^, it is possible to calibrate the measurements [50]. Briefly, Fura-2 AM (1 µM) was added to NHEK- or TRPV3-overexpressing HEK 293T cells in normal Tyrode (NT) solution comprising 145 mM NaCl, 3.6 mM KCl, 1.3 mM CaCl_2_, 1 mM MgCl_2_, 5 mM glucose, 20 mM sorbitol, and 10 mM HEPES (pH 7.4, adjusted with NaOH) and incubated for 30 min at 37 °C. The loaded cells were centrifuged and resuspended in NT solution. G573S- or G573C-transfected cells were prepared in NT solution without calcium using a similar protocol. Samples were excited using a wavelength of 380 nm for 20 ms, followed by 340 nm for 100 ms, and fluorescence at 510 nm was recorded. Recorded data were then analyzed using NIS-Element AR Version 5.00.00 (Nikon Instruments, Melville, NY, USA).

The change in the fluorescence ratio when the cells were excited at 340 nm and 380 nm (ΔF_340/380_) during the measurements was calculated by subtracting the ratio obtained at the beginning of the experiment from each subsequent ratio obtained at different time points. Since higher F_340/380_ implies a higher Ca^2+^ level, these values allow the assessment of the relative changes in the intracellular Ca^2+^ level over time.

### 4.6. Viability Assay

The cell counting kit-8 (CCK8; Dojindo Laboratories, Kumamoto, Japan) was employed for viability assay. Keratinocytes (10^4^ cells/well) plated in 96-well plates were incubated for about one day before exposing them to alpha-mangostin for 24 h. Transiently transfected HEK 293T cells (5 × 10^4^ cells/well) in 96-well plates were plated and exposed to carvacrol, 74a, or alpha-mangostin 6 h after transfection, followed by 24 h incubation. The optical density (O.D., 450 nm) of each well was measured after incubating the cells with CCK-8 solution (10 μM) for 2 h. The normalized O.D. value of each well compared with the mock group was used to determine cell viability.

### 4.7. Cytokine Assay

Keratinocytes were first plated in 24-well plates (5 × 10^4^ cells/well) for 24 h; then, 300 µM carvacrol, 10 µM 74a, or 1 µM alpha-mangostin were added, followed by a 24 h incubation before collecting the cell culture supernatant to measure the cytokine content. Assays were performed using an ELISA kit (KOMA Biotech Co. Ltd., Seoul, Korea) following the supplier’s guidelines.

### 4.8. Statistical Analysis

All results in this study are shown as means ± standard error of the mean. One-way analysis of variance (ANOVA) combined with Bonferroni’s post hoc comparison was applied for multiple comparisons as appropriate. Statistical analyses were conducted using Origin 2021b (Microcal) and GraphPad Prism 8.2.1 (GraphPad Software, San Diego, CA, USA) software; *p*-values lower than 0.05 were considered statistically significant.

## 5. Conclusions

We identified alpha-mangostin as a natural TRPV3 inhibitor that can alleviate cytotoxicity and suppress the release of pro-inflammatory cytokines caused by excessive TRPV3 activity, which might be a potential therapeutic strategy for abnormal skin conditions. The effects of alpha-mangostin on various human skin pathological conditions and skin disease mouse models, as well as the molecular mechanism of its inhibitory effect on the TRPV3 channel, should be further investigated. Our results suggest that alpha-mangostin could be developed as a potential drug for treating various skin inflammatory conditions.

## Figures and Tables

**Table 1:** Natural TRPV3 inhibitors.

Natural Compound	Chemical Structure	Channel Inhibited	IC_50_ (µM)
Alpha-mangostin	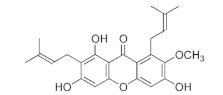	TRPV3	0.077 ± 0.013
		TRPV1	0.43 ± 0.27
		TRPV4	8.470 ± 1.725
		TRPA1	5.092 ± 2.213
Citrusinine II [34]	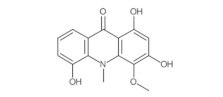	TRPV3	12.43 ± 1.86
Coumarin osthole [35,36]	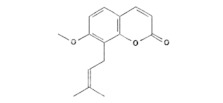	TRPV3	37.0 ± 1.9
		TRPV1	Not available
Isochlorogenic acid A [37]	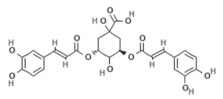	TRPV3	2.7 ± 1.3
Isochlorogenic acid B [37]	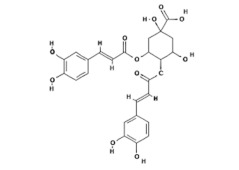	TRPV3	0.9 ± 0.3
Forsythoside B [38]	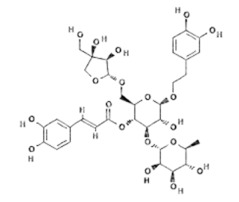	TRPV3	6.7 ± 0.7
Verbascoside [14]	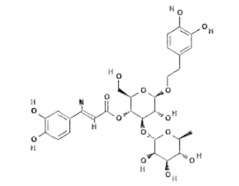	TRPV3	14.1 ± 3.3
Monanchomycalin B [39]	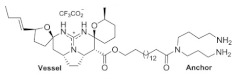	TRPV3	3.25 ± 0.6
		TRPV1	6.02 ± 0.36
		TRPV2	2.84 ± 1.01
Pulchranin A [40,41]	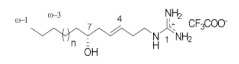 n = 3	TRPV3	71.8 ± 9.4
		TRPV1	27.5 ± 1.4
		TRPA1	174.2 ± 7.4
Pulchranin B [40]	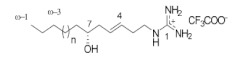 n = 2	TRPV3	117.9 ± 11.8
		TRPV1	95.4 ± 13.1
		TRPA1	>200
Pulchranin C [40]	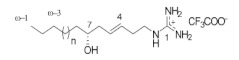 n = 1	TRPV3	>200
		TRPV1	182.7 ± 27.0
		TRPA1	>200

**Table 2:** Composition of solutions used for whole-cell patch-clamp recording.

Ion Channel	Bath Solution (in mM)	Pipette Solution (in mM)
TRPV3	139 NaCl, 5 KCl, 1.8 BaCl_2_, 2 MgCl_2_, 10 glucose, 10 sorbitol, and 10 HEPES (pH 7.4, adjusted with NaOH)	120 CsCl, 2 MgCl_2_, 3 Mg-ATP, 10 EGTA, 10 HEPES, and 3.15 CaCl_2_ (equivalent to 150 nM free Ca^2+^, calculated by the free Webmaxc software) (pH 7.2, adjusted with CsOH)
TRPV3 mutants		120 CsCl, 2 MgCl_2_, 3 Mg-ATP, 10 EGTA, and 10 HEPES (pH 7.2, adjusted with CsOH)
TRPV4	145 NaCl, 3.6 KCl, 10 HEPES, 5 glucose, 1 MgCl_2_, and 1.3 CaCl_2_ (pH 7.4, adjusted with NaOH)	140 CsCl, 1 MgCl_2_, 5 HEPES, 10 EGTA, and 3 Mg-ATP (pH 7.2, titrated by CsOH)
TRPA1		150 CsCl, 10 BAPTA, 10 HEPES, and 1 MgCl_2_ (pH 7.2, adjusted by CsOH)

## Data Availability

The data, analytic methods, and study materials that support the findings of this study are available from the corresponding author upon reasonable request.

## Supplementary Materials

The supporting information can be downloaded at: https://www.mdpi.com/article/10.3390/ijms241612930/s1.

## Abbreviations

α-MG, alpha-mangostin; HEK, human embryonic kidney; IL, interleukin; NHEK, normal human epidermal keratinocyte; TNF-α, tumor necrosis factor-alpha.

## References

[1] Lee S.E., Lee S.H. (2018). Skin barrier and calcium. Ann. Dermatol..

[2] Luo J., Hu H. (2014). Thermally activated TRPV3 channels. Curr. Top. Membr..

[3] Wang Y., Li H., Xue C., Chen H., Xue Y., Zhao F., Zhu M.X., Cao Z. (2021). TRPV3 enhances skin keratinocyte proliferation through EGFR-dependent signaling pathways. Cell Biol. Toxicol..

[4] Cheng X., Jin J., Hu L., Shen D., Dong X.P., Samie M.A., Knoff J., Eisinger B., Liu M.L., Huang S.M. (2010). TRP channel regulates EGFR signaling in hair morphogenesis and skin barrier formation. Cell.

[5] Aijima R., Wang B., Takao T., Mihara H., Kashio M., Ohsaki Y., Zhang J.Q., Mizuno A., Suzuki M., Yamashita Y. (2015). The thermosensitive TRPV3 channel contributes to rapid wound healing in oral epithelia. FASEB J..

[6] Borbíró I., Lisztes E., Tóth B.I., Czifra G., Oláh A., Szöllosi A.G., Szentandrássy N., Nánási P.P., Péter Z., Paus R. (2011). Activation of transient receptor potential vanilloid-3 inhibits human hair growth. J. Investig. Dermatol..

[7] Singh A.K., McGoldrick L.L., Demirkhanyan L., Leslie M., Zakharian E., Sobolevsky A.I. (2019). Structural basis of temperature sensation by the TRP channel TRPV3. Nat. Struct. Mol. Biol..

[8] Moore C., Gupta R., Jordt S.E., Chen Y., Liedtke W.B. (2018). Regulation of pain and itch by TRP channels. Neurosci. Bull..

[9] Asakawa M., Yoshioka T., Matsutani T., Hikita I., Suzuki M., Oshima I., Tsukahara K., Arimura A., Horikawa T., Hirasawa T. (2006). Association of a mutation in TRPV3 with defective hair growth in rodents. J. Investig. Dermatol..

[10] Yoshioka T., Imura K., Asakawa M., Suzuki M., Oshima I., Hirasawa T., Sakata T., Horikawa T., Arimura A. (2009). Impact of the Gly573Ser substitution in TRPV3 on the development of allergic and pruritic dermatitis in mice. J. Investig. Dermatol..

[11] Lin Z., Chen Q., Lee M., Cao X., Zhang J., Ma D., Chen L., Hu X., Wang H., Wang X. (2012). Exome sequencing reveals mutations in TRPV3 as a cause of Olmsted syndrome. Am. J. Hum. Genet..

[12] Qu Y., Wang G., Sun X., Wang K. (2019). Inhibition of the warm temperature-activated Ca^2+^-permeable transient receptor potential vanilloid TRPV3 channel attenuates atopic dermatitis. Mol. Pharmacol..

[13] Sonkoly E., Muller A., Lauerma A.I., Pivarcsi A., Soto H., Kemeny L., Alenius H., Dieu-Nosjean M.C., Meller S., Rieker J. (2006). IL-31: A new link between T cells and pruritus in atopic skin inflammation. J. Allergy Clin. Immunol..

[14] Sun X., Qi H., Wu H., Qu Y., Wang K. (2020). Antipruritic and anti-inflammatory effects of natural verbascoside through selective inhibition of temperature-sensitive Ca^2+^-permeable TRPV3 channel. J. Dermatol. Sci..

[15] Szöllősi A.G., Vasas N., Angyal Á., Kistamás K., Nánási P.P., Mihály J., Béke G., Herczeg-Lisztes E., Szegedi A., Kawada N. (2018). Activation of TRPV3 regulates inflammatory actions of human epidermal keratinocytes. J. Investig. Dermatol..

[16] Zhao J., Munanairi A., Liu X.Y., Zhang J., Hu L., Hu M., Bu D., Liu L., Xie Z., Kim B.S. (2020). PAR2 mediates itch via TRPV3 signaling in keratinocytes. J. Investig. Dermatol..

[17] Yamamoto-Kasai E., Imura K., Yasui K., Shichijou M., Oshima I., Hirasawa T., Sakata T., Yoshioka T. (2012). TRPV3 as a therapeutic target for itch. J. Investig. Dermatol..

[18] Dias D.A., Urban S., Roessner U. (2012). A historical overview of natural products in drug discovery. Metabolites.

[19] Thomford N.E., Senthebane D.A., Rowe A., Munro D., Seele P., Maroyi A., Dzobo K. (2018). Natural Products for Drug Discovery in the 21st Century: Innovations for Novel Drug Discovery. Int. J. Mol. Sci..

[20] Ibrahim M.Y., Hashim N.M., Mariod A.A., Mohan S., Abdulla M.A., Abdelwahab S.I., Arbab I.A. (2016). α-mangostin from *Garcinia mangostana* Linn: An updated review of its pharmacological properties. Arab. J. Chem..

[21] Mohan S., Syam S., Abdelwahab S.I., Thangavel N. (2018). An anti-inflammatory molecular mechanism of action of α-mangostin, the major xanthone from the pericarp of *Garcinia mangostana*: An in silico, in vitro and in vivo approach. Food Funct..

[22] Zhou S., Yotsumoto H., Tian Y., Sakamoto K. (2021). α-mangostin suppressed melanogenesis in B16F10 murine melanoma cells through GSK3β and ERK signaling pathway. Biochem. Biophys. Rep..

[23] Higuchi H., Tanaka A., Nishikawa S., Oida K., Matsuda A., Jung K., Amagai Y., Matsuda H. (2013). Suppressive effect of mangosteen rind extract on the spontaneous development of atopic dermatitis in NC/Tnd mice. J. Dermatol..

[24] Kondo S., Kono T., Sauder D.N., McKenzie R.C. (1993). IL-8 gene expression and production in human keratinocytes and their modulation by UVB. J. Investig. Dermatol..

[25] Xu N., Deng W., He G., Gan X., Gao S., Chen Y., Gao Y., Xu K., Qi J., Lin H. (2018). Alpha- and gamma-mangostins exhibit anti-acne activities via multiple mechanisms. Immunopharmacol. Immunotoxicol..

[26] Nilius B., Bíró T. (2013). TRPV3: A ‘more than skinny’ channel. Exp. Dermatol..

[27] Xu H., Delling M., Jun J.C., Clapham D.E. (2006). Oregano, thyme and clove-derived flavors and skin sensitizers activate specific TRP channels. Nat. Neurosci..

[28] Humeau M., Boniface K., Bodet C. (2022). Cytokine-mediated crosstalk between keratinocytes and T cells in atopic dermatitis. Front. Immunol.

[29] Su W., Qiao X., Wang W., He S., Liang K., Hong X. (2023). TRPV3: Structure, diseases and modulators. Molecules.

[30] Sulk M., Seeliger S., Aubert J., Schwab V.D., Cevikbas F., Rivier M., Nowak P., Voegel J.J., Buddenkotte J., Steinhoff M. (2012). Distribution and expression of non-neuronal transient receptor potential (TRPV) ion channels in rosacea. J. Investig. Dermatol..

[31] Scott V.E., Patel H., Wetter J., Edlmayer R., Neelands T., Miller L., Huang S., Gauld S., Todorovic V., Gomtsian A. (2016). 534 Defining a mechanistic link between TRPV3 activity and psoriasis through IL-1α and EGFR signaling pathways. J. Investig. Dermatol..

[32] Um J.Y., Kim H.B., Kim J.C., Park J.S., Lee S.Y., Chung B.Y., Park C.W., Kim H.O. (2022). TRPV3 and Itch: The Role of TRPV3 in Chronic pruritus according to Clinical and Experimental Evidence. Int. J. Mol. Sci..

[33] Kim S.E., Yin M.Z., Roh J.W., Kim H.J., Choi S.W., Wainger B.J., Kim W.K., Kim S.J., Nam J.H. (2023). Multi-target modulation of ion channels underlying the analgesic effects of α-mangostin in dorsal root ganglion neurons. Phytomedicine.

[34] Han Y., Luo A., Kamau P.M., Takomthong P., Hu J., Boonyarat C., Luo L., Lai R. (2021). A plant-derived TRPV3 inhibitor suppresses pain and itch. Br. J. Pharmacol..

[35] Sun X.Y., Sun L.L., Qi H., Gao Q., Wang G.X., Wei N.N., Wang K. (2018). Antipruritic effect of natural coumarin osthole through selective inhibition of thermosensitive TRPV3 channel in the skin. Mol. Pharmacol..

[36] Yang N.N., Shi H., Yu G., Wang C.M., Zhu C., Yang Y., Yuan X.L., Tang M., Wang Z.L., Gegen T. (2016). Osthole inhibits histamine-dependent itch via modulating TRPV1 activity. Sci. Rep..

[37] Qi H., Shi Y., Wu H., Niu C., Sun X., Wang K. (2022). Inhibition of temperature-sensitive TRPV3 channel by two natural isochlorogenic acid isomers for alleviation of dermatitis and chronic pruritus. Acta Pharm. Sin. B.

[38] Zhang H., Sun X., Qi H., Ma Q., Zhou Q., Wang W., Wang K. (2019). Pharmacological inhibition of the temperature-sensitive and Ca2+-permeable transient receptor potential vanilloid TRPV3 channel by natural forsythoside B attenuates pruritus and cytotoxicity of keratinocytes. J. Pharmacol. Exp. Ther..

[39] Korolkova Y., Makarieva T., Tabakmakher K., Shubina L., Kudryashova E., Andreev Y., Mosharova I., Lee H.S., Lee Y.J., Kozlov S. (2017). Marine cyclic guanidine alkaloids Monanchomycalin B and Urupocidin A act as inhibitors of TRPV1, TRPV2 and TRPV3, but not TRPA1 receptors. Mar. Drugs.

[40] Makarieva T.N., Ogurtsova E.K., Korolkova Y.V., Andreev Y.A., Mosharova I.V., Tabakmakher K.M., Guzii A.G., Denisenko V.A., Dmitrenok P.S., Lee H.S. (2013). Pulchranins B and C, new acyclic guanidine alkaloids from the Far-Eastern marine sponge Monanchora pulchra. Nat. Prod. Commun..

[41] Guzii A.G., Makarieva T.N., Korolkova Y.V., Andreev Y.A., Mosharova I.V., Tabakmaher K.M., Denisenko V.A., Dmitrenok P.S., Ogurtsova E.K., Antonov A.S. (2013). Pulchranin A, isolated from the Far-Eastern marine sponge, *Monanchora. pulchra*: The first marine non-peptide inhibitor of TRPV-1 channels. Tetrahedron. Lett..

[42] Larkin C., Chen W., Szabó I.L., Shan C., Dajnoki Z., Szegedi A., Buhl T., Fan Y., O’Neill S., Walls D. (2021). Novel insights into the TRPV3-mediated itch in atopic dermatitis. J. Allergy Clin. Immunol..

[43] Pan-In P., Wongsomboon A., Kokpol C., Chaichanawongsaroj N., Wanichwecharungruang S. (2015). Depositing α-mangostin nanoparticles to sebaceous gland area for acne treatment. J. Pharmacol. Sci..

[44] Tan Y.F., Koay Y.S., Zulkifli R.M., Abdul Hamid M. (2022). In vitro hair growth and hair tanning activities of mangosteen pericarp extract on hair dermal papilla cells. J. Herb. Med..

[45] Nam J.H., Jung H.W., Chin Y.W., Yang W.M., Bae H.S., Kim W.K. (2017). Spirodela polyrhiza extract modulates the activation of atopic dermatitis-related ion channels, Orai1 and TRPV3, and inhibits mast cell degranulation. Pharm. Biol..

[46] Kim H.J., Nam Y.R., Nam J.H. (2018). Flos Magnoliae inhibits chloride secretion via ANO1 inhibition in Calu-3 cells. Am. J. Chin. Med..

[47] Woo J., Kim H.J., Nam Y.R., Kim Y.K., Lee E.J., Choi I., Kim S.J., Lee W., Nam J.H. (2018). Mitochondrial dysfunction reduces the activity of KIR2.1 K^+^ channel in myoblasts via impaired oxidative phosphorylation. Korean J. Physiol. Pharmacol..

[48] Woo J.H., Nam D.Y., Kim H.J., Hong P.T.L., Kim W.K., Nam J.H. (2021). Nootkatol prevents ultraviolet radiation-induced photoaging via ORAI1 and TRPV1 inhibition in melanocytes and keratinocytes. Korean J. Physiol. Pharmacol..

[49] Martínez M., Martínez N.A., Silva W.I. (2017). Measurement of the intracellular calcium concentration with Fura-2 AM using a fluorescence plate reader. Bio Protoc..

[50] Tinning P.W., Franssen A.J.P.M., Hridi S.U., Bushell T.J., McConnell G.A. (2018). 340/380 nm light-emitting diode illuminator for Fura-2 AM ratiometric Ca^2+^ imaging of live cells with better than 5 nM precision. J. Microsc..

