# Supplementary material for: Alpha-Mangostin: A Potent Inhibitor of TRPV3 and Pro-Inflammatory Cytokine Secretion in Keratinocytes
Source: Int J Mol Sci. 2023 Aug 18;24(16):12930. doi: 10.3390/ijms241612930 (PMC10455244; doi:10.3390/ijms241612930)
Supplement: Supplementary file 1 [file ijms-24-12930-s001.zip › ijms-2569864-supplementary.pdf]

Supplementary

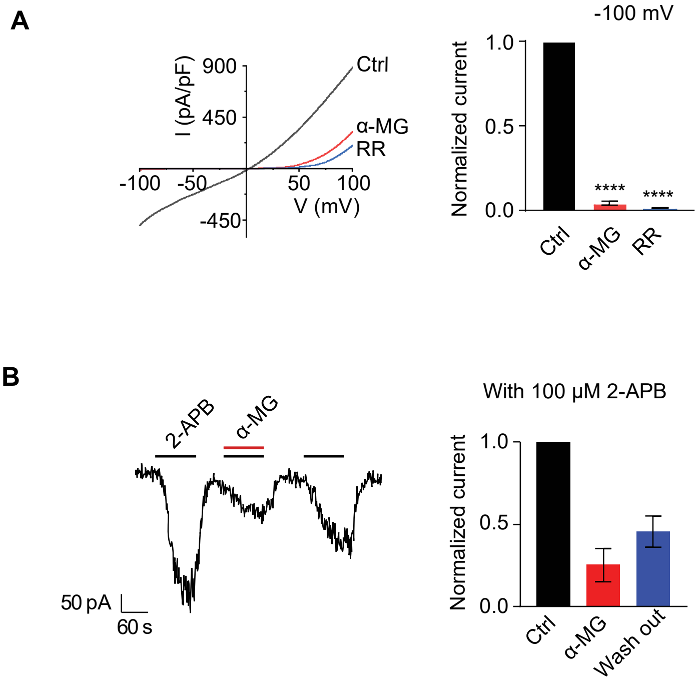

Figure S1. (A) Alpha-mangostin almost completely inhibited 2-APB-evoked TRPV3 current at 10 μM. The left panel shows the current-voltage relationship and the right panel shows the summary of the normalized current at −100 mV. Currents were elicited with ramp pulses from −100 to +100 mV over 1 s, which was repetitively applied every 20 s, and the holding potential was set at 0 mV. The channel was activated using 100 μM 2-APB and subsequently perfused with solutions containing 10 μM alpha-mangostin with 100 μM 2-APB. A solution containing 10 μM ruthenium red (RR, a TRP channel inhibitor) and 100 μM 2-APB was perfused at the end. Currents were normalized to the maximum response to 2-APB at −100 mV. (B) The inhibitory effect of alpha-mangostin on TRPV3 current was partly reversible. The left panel shows the representative traces recorded at– 60 mV holding potential, and the right panel shows the summary of normalized current at – 60 mV. Currents were normalized to the maximum response to 2-APB at – 60 mV. Data are presented as means ± SEM.
